# The Association Between Moral Courage, Psychological Resilience, and Workplace Violence Among Emergency Department Nurses in China: A Structural Equation Model

**DOI:** 10.1155/jonm/5540407

**Published:** 2026-07-11

**Authors:** Kai Liu, XinYue Zhang, XiangHong Sun, Ling Fan

**Affiliations:** ^1^ Department of Nursing, Shengjing Hospital of China Medical University, Shenyang, China, cmu.edu.cn; ^2^ School of Nursing, China Medical University, Shenyang, China, cmu.edu.tw; ^3^ Clinical Skills Practical Teaching Center, Shengjing Hospital of China Medical University, Shenyang, China, cmu.edu.cn

**Keywords:** emergency nurse, moral courage, psychological resilience, structural equation model, workplace violence

## Abstract

**Aim:**

The objective of this study is to examine the association between moral courage, psychological resilience, and the occurrence of workplace violence among emergency department nurses.

**Background:**

Workplace violence represents a major occupational hazard in healthcare institutions worldwide. Moral courage refers to nurses’ ability to make principled decisions when facing ethical conflicts, thereby avoiding moral distress. Psychological resilience is defined as an individual’s positive internal capacity to cope with adversity and stress. Such violent incidents severely undermine nurses’ physical and mental well‐being, while moral courage plays a pivotal role in enhancing nurses’ psychological resilience and improving their mental health. However, the underlying mechanism through which moral courage influences nurses’ experiences of workplace violence remains unexplored.

**Methods:**

This study surveyed 325 emergency nurses in Liaoning Province, China, using the Nurses’ General Information Form, the Nurses’ Moral Courage Scale, the Psychological Resilience Scale, and the Workplace Violence Scale. Descriptive analysis, correlation analysis, and analysis of influencing factors were conducted using SPSS 27.0, while AMOS 27.0 was employed for the verification and analysis of structural equation modeling.

**Results:**

The final model explained 35.0% of the total variance in workplace violence among emergency department nurses. Moral courage had a positive impact on psychological resilience, with a path coefficient *β* = 0.710 (*t* = 15.347, *p* < 0.001). Psychological resilience negatively predicted workplace violence, with a path coefficient *β* = −0.210 (*t* = −2.466, *p* = 0.014). Similarly, moral courage negatively predicted workplace violence, with a path coefficient *β* = −0.231 (*t* = −2.709, *p* = 0.007). Psychological resilience played a partial mediating role between moral courage and workplace violence, accounting for 35.89% of the mediating effect.

**Conclusions:**

This study employed structural equation modeling and found a significant negative correlation between moral courage and workplace violence, as well as a partial mediating effect of psychological resilience in the relationship between moral courage and workplace violence.

**Implications for Nursing Management:**

In the future, training measures can be developed from enhancing nurses’ moral courage and psychological resilience, and constantly optimize and improve the existing interventions.

**Patient or Public Contribution:**

No patient or public contribution.

## 1. Introduction

Workplace violence (WPV) is defined as incidents where individuals or personnel on duty experience verbal or physical violence, abuse, threats, or attacks in work‐related environments, posing explicit or implicit challenges to employees’ safety, well‐being, and even health [1, 2]. According to a report by the World Health Organization (WHO), nearly 50% of healthcare workers worldwide have experienced at least one incident of WPV during their careers [3, 4]. Emergency departments, as special units within hospitals, shoulder the heavy responsibility of treating a large number of critically ill patients. Due to the high frequency of emergencies and the high mobility of patients, emergency department staff are not only the main force in medical treatment but also the group that has the closest contact with patients and their families, making them frequent targets of negative emotional outlet from others [5]. Research conducted abroad has demonstrated that the prevalence of WPV among emergency nurses can be as high as 77%–91.9% [6, 7]. Similarly, in China, the incidence rate of WPV among emergency nurses remains notably high, ranging from 72.5% to 91.5% [8–10]. After the pandemic, this phenomenon has further intensified [3]. A systematic review published in 2026 showed that the rate of verbal abuse among healthcare workers in emergency and acute care settings remains above 75%, while formally reported violent incidents account for less than 25% [11]. WPV has emerged as a major occupational hazard in healthcare institutions globally [12]. This hazard exerts tremendous pressure on the physical and mental health of emergency nurses [9]. A theoretical framework study on WPV among emergency nurses indicates that this issue arises from the interplay of multiple factors, including patients, staff, organizations, and environmental influences [13]. Therefore, to mitigate the negative impacts of WPV, it is essential to investigate its underlying factors in order to develop effective intervention strategies.

When violent incidents occur, nurses frequently encounter challenging dilemmas, such as whether to strictly adhere to established protocols or to prioritize injury treatment for the aggressive patient. To effectively address such dilemmas, nurses require moral courage [14]. Moral courage is defined as the ability of nurses to make correct decisions based on their own principles when confronted with ethical conflicts, thereby avoiding moral distress [15]. Relevant studies have indicated that qualities such as overcoming fear, which nurses possess during emergencies, including violent incidents, are indispensable components of moral courage [16]. Nurses with higher levels of moral courage tend to adopt proactive coping strategies when encountering violent situations, whereas those with lower levels of moral courage are more prone to falling into negative emotional responses [17].

Notably, although moral courage is an intrinsic personal quality [18], it is also shaped by organizational culture [19]. The emergency department, as a high‐incidence area for WPV, is influenced by management’s zero‐tolerance policies toward violence, the ethical climate, and the support and protection mechanisms provided to nurses after reporting violent incidents—all of which shape nurses’ behavioral choices when facing conflicts. A supportive organizational culture can encourage nurses to translate their intrinsic moral courage into proactive strategies for responding to violent incidents [17, 20]; conversely, when organizations lack effective regulatory and support systems, nurses’ moral courage may be suppressed, thereby undermining their efficacy and behavioral expression in responding to violence. Moral courage is also considered to be developable and improvable through instruction or learning [14]. Therefore, in nursing practice, implementing targeted training programs or improving the organizational environment can effectively enhance nurses’ moral courage, offering a novel perspective for addressing violent incidents [21]. To date, no study has explicitly elucidated the direct relationship between moral courage and WPV.

An international study [22] has demonstrated that there is a correlation between the level of moral courage and the level of psychological resilience among nursing students. Research by Abdollahi et al. [23] also indicates that nurses’ level of moral courage influences their level of psychological resilience—specifically, the higher the level of moral courage, the higher the level of psychological resilience. Psychological resilience refers to an individual’s positive internal coping ability when facing adversity and difficulties [24]. Psychological resilience is a relatively modifiable intrinsic trait of individuals [25], which can serve as an effective strategy to cope with and reduce the frequency of violent incidents [26]. As a positive psychological resource, psychological resilience can effectively mitigate the negative impacts of WPV and is an important protective factor for individual physical and mental health [27]. Studies have shown that there is a negative correlation between the psychological resilience scores of emergency department nurses and WPV [28, 29].

However, the relationship between moral courage and WPV, as well as whether psychological resilience mediates between moral courage and WPV, remains unclear. In general, there is a lack of research explicitly exploring the relationship between WPV, psychological resilience, and moral courage. Therefore, this study constructs a mediation model with moral courage as the independent variable, psychological resilience as the mediating variable, and WPV as the dependent variable, proposing the following hypotheses:

 Hypothesis 1: Moral courage is negatively correlated with WPV and negatively predicts WPV;
 Hypothesis 2: Psychological resilience mediates between moral courage and exposure to WPV.

## 2. Methods

### 2.1. Study Design and Participants

This is a multicenter cross‐sectional study and here reported according to the guidelines for STrengthening the Reporting of OBservational studies in Epidemiology (STROBE). Using convenience sampling, emergency department nurses from five tertiary‐level hospitals in Liaoning Province were recruited as the study subjects from October 2024 to December 2024, based on the following inclusion and exclusion criteria.

Inclusion criteria are as follows: (1) currently registered nurses, with ≥ 1 year of experience in emergency nursing; (2) nurses who provided informed consent and voluntarily participated. Exclusion criteria are as follows: (1) nurses in residency training, interns, nurses on maternity leave or sick leave; (2) nurses who were transferred out of the emergency department or rotated into the emergency department for ≤ 1 year during the study period. The sample size was estimated based on the Kendall’s [30] criterion for sample size calculation in multifactor analysis design, with the sample size being 5 to 10 times the number of independent variables. Additionally, a 20% sample loss rate was considered. The sample size ranged from 150 to 300 cases. Ultimately, 325 emergency department nurses were surveyed, with 304 cases actually included in the analysis.

### 2.2. Measures

#### 2.2.1. Demographic Characteristics

A demographic data sheet for nurses was self‐designed by the researcher based on a literature review and expert opinions. It includes demographics such as gender, age, educational background, professional title, years of work experience, employment status, marital status, working hours, and monthly income.

#### 2.2.2. WPV

The Workplace Violence Scale (WVS), developed by Wang in 2006, investigates the types and frequency of WPV experienced by individuals from patients and their families [31]. The scale consists of five items: physical attack, verbal abuse, threatening intimidation, verbal sexual harassment, and physical sexual harassment. It employs a Likert 4‐point rating scale (0 = “never experienced violence,” 1 = “experienced once,” 2 = “experienced 2‐3 times,” 3 = “experienced more than 4 times”). The total score of the scale ranges from 0 to 15, with higher scores indicating more exposure to violence. The Cronbach’s *α* coefficient of this scale is 0.92. In this study, the Cronbach’s *α* coefficient of the scale was 0.827.

#### 2.2.3. Psychological Resilience

The Connor‐Davidson Resilience Scale (CD‐RISC), developed by American scholars Connor and Davidson in 2003 [32], was translated into Chinese by Yu and Zhang [33] in 2007. The scale consists of 25 items across three dimensions: Tenacity (13 items), Optimism (8 items), and Self‐reliance (4 items). Specifically, Tenacity includes items 11, 12, 13, 14, 15, 16, 17, 18, 19, 20, 21, 22, and 23; Self‐reliance includes items 1, 5, 7, 8, 9, 10, 24, and 25; and Optimism includes items 2, 3, 4, and 6. The scale employs a Likert 5‐point rating system ranging from “Never” (0 points) to “Always” (4 points), with a total score ranging from 0 to 100. Scores of 0–56 indicate a low level of resilience, 57 to 70 indicate a medium level, and 71 to 100 indicate a high level [34]. The Cronbach’s *α* coefficient of the scale is 0.910. In this study, the Cronbach’s *α* coefficient of the scale was 0.928.

#### 2.2.4. Moral Courage

The Nurses’ Moral Courage Scale (NMCS) was developed by Numminen et al. in 2018 [35] and Wang et al. in 2019 validated its reliability and validity [36]. The scale consists of 21 items across four dimensions: Ethical Conduct (7 items: 1, 4, 9, 11, 12, 19, 21), Commitment to Quality Care (5 items: 5, 8, 14, 16, 18), Empathy and Being Truly Present with Patients (5 items: 2, 10, 15, 17, 20), and Moral Responsibility (4 items: 3, 6, 7, 13). It employs a Likert 5‐point scale ranging from “Completely Disagree” to “Completely Agree” (1–5 points). The total score of the scale ranges from 21 to 105, with a higher score indicating a higher level of moral courage among nurses. The Cronbach’s *α* coefficient of the scale is 0.905. In this study, the Cronbach’s *α* coefficient of the scale was 0.950.

### 2.3. Data Collection and Quality Control

The researchers first contacted the head nurses of the emergency departments of each sampling unit (five tertiary hospitals) to explain the study purpose, content, and significance, and obtained permission from the departments and nursing administrations. Subsequently, the head nurses distributed the QR code and link to the questionnaire generated by Wenjuanxing (an online survey platform) in the departmental work groups. Nurses who met the inclusion and exclusion criteria were invited to complete the questionnaire. The head nurses informed the nurses that participation was voluntary and anonymous, and that all data would be used solely for academic research, not involving any performance appraisal. Participants entered the questionnaire page by clicking the link or scanning the QR code and completed it independently and anonymously. Before filling out the questionnaire, they were required to check a box indicating informed consent. The system was set to allow only one response per IP address or WeChat account to avoid duplicate submissions. The questionnaire took approximately 15–20 min to complete. The researchers monitored the response status in real time through the backend and excluded questionnaires that were incomplete or logically inconsistent.

### 2.4. Statistical Analysis

Data analysis was conducted using SPSS 27.0 software. For measurement data, if normally distributed, they were described using mean ± standard deviation (±SD); if non‐normally distributed, they were described using median and interquartile range. Categorical data were described using frequency (*n*) and proportion (%). Spearman correlation analysis was employed to explore the relationships among WPV, psychological resilience, and moral courage. AMOS 27.0 was utilized to verify and revise the model, analyzing the mediating role of psychological resilience between moral courage and WPV. The significance of the mediating effect was tested using the bootstrap method. A two‐tailed test was applied with a significance level of *α* = 0.05.

### 2.5. Ethics Approval

This study was approved by the Ethics Committee of Shengjing Hospital of China Medical University in Shenyang (2024PS1782K). All data were collected anonymously and kept confidential.

## 3. Results

### 3.1. Baseline Characteristics of Emergency Nurses

A total of 325 questionnaires were distributed in this study, and 304 emergency department nurses in Liaoning Province were included after excluding invalid questionnaires, with a valid rate of 93.5%. Among them, there were 84 males (27.6%) and 220 females (72.4%). Other details are shown in Table 1.

**TABLE 1:** The baseline characteristics of emergency nurses (*n* = 304).

Feature		Number	Ratio (%)
Gender	Male	84	27.6
	Female	220	72.4

Age	20–25	32	10.5
	26–30	36	11.8
	31–35	65	21.4
	36–40	120	39.5
	41–45	34	11.2
	> 45	17	5.6

Education	College degree or below	41	13.5
	Bachelor’s degree or above	263	86.5

Marital status	Married	226	74.3
	Unmarried	73	24.0
	Divorced	5	1.6

Having children	Yes	214	70.4
	No	90	29.6

Employment status	Permanent	42	13.8
	On contract	262	86.2

Working experience	1–2 years	25	8.2
	3–5 years	25	8.2
	6–10 years	49	16.1
	10–15 years	128	42.1
	Over 15 years	77	25.3

Working experience in emergency	1–2 years	61	20.1
	3–5 years	36	11.8
	6–10 years	48	15.8
	10–15 years	108	35.5
	Over 15 years	51	16.8

Professional role	Nurse	33	10.9
	Nursing technician	151	49.7
	Senior nursing technician	112	36.8
	Deputy chef nursing technician	8	2.6

Working area	Green area	91	29.9
	Yellow area	38	12.5
	Red area	89	29.3
	EICU	80	26.3
	Pre‐emergency	6	2.0

Duty	Day shift leader	25	8.2
	Day shift nurse	105	34.5
	Night shift leader	36	11.8
	Night shift nurse	138	45.4

Frequency of night shift (per month)	0	56	18.4
	1–3	5	1.6
	3–5	45	14.8
	5–8	198	65.1

Income	1000–3000	20	6.6
	3000–5000	74	24.3
	5000–10000	133	43.8
	> 10,000	77	25.3

Presence or absence of family accompaniment	Yes	243	79.9
	No	61	20.1

Nurse resource	Always enough	151	49.7
	Sometimes enough	135	44.4
	Seldom enough	15	4.9
	Never enough	3	1.0

*Note:* In the emergency work area, patients are self‐ambulatory without severe trauma; the yellow area is patients with potential death risk of serious injury; the red area is critically ill patients who must be treated immediately; and the EICU is an intensive care unit.

### 3.2. Scores of WPV, Moral Courage, and Resilience, and Their Dimensions Among Emergency Nurses

The findings of this study indicate that the prevalence of WPV among emergency department nurses is categorized within the upper‐middle range, their psychological resilience was assessed as moderate, and their moral courage was found to be above the average level. Among the 304 nurses, 184 had experienced at least one instance of WPV in the past year, with an incidence rate of 60.5%. According to the classification of WPV, the incidence rates of various types of violence among emergency department nurses, from highest to lowest, were verbal abuse (58.9%), intimidation and threats (36.5%), physical assault (26.6%), verbal harassment (17.8%), and physical harassment (9.9%). For details, see Tables 2 and 3.

**TABLE 2:** Scores of WPV, moral courage, and resilience, and their dimensions among emergency nurses (*n* = 304).

Context	Item number	Total score ($\overset{¯}{x}$ ± SD)	Mean score per item ($\overset{¯}{x}$ ± SD)
Workplace violence	5	3.20 ± 2.56	0.64 ± 0.51
Moral courage	21	81.08 ± 17.16	3.86 ± 0.81
Morality	7	22.36 ± 5.40	3.80 ± 0.85
Empathy	5	19.93 ± 4.29	3.98 ± 0.85
Moral responsibility	4	15.82 ± 3.56	3.95 ± 0.89
Proposal of good care	5	18.69 ± 4.55	3.71 ± 0.91
Psychological resilience	25	67.94 ± 20.47	3.86 ± 0.81
Self‐improvement	8	23.03 ± 6.71	2.88 ± 0.83
Optimism	4	10.30 ± 3.58	2.57 ± 0.89
Strength	13	34.59 ± 11.33	2.66 ± 0.87

**TABLE 3:** The type and frequency of violence issues suffered by emergency nurses within 1 year (*n* = 304).

The type of violence	0 (%)	1 time (%)	≥ 1 times (%)	2∼3 times (%)	≥ 4 times (%)
Verbal abuse	125 (41.1)	73 (24.0)	179 (58.9)	64 (21.1)	42 (13.8)
Intimidation and threats	193 (63.5)	59 (19.4)	111 (36.5)	31 (10.2)	21 (6.9)
Physical assault	223 (73.4)	41 (13.5)	81 (26.6)	27 (8.9)	13 (4.3)
Verbal harassment	250 (82.2)	26 (8.6)	54 (17.8)	18 (5.9)	10 (3.3)
Physical harassment	274 (90.1)	19 (6.3)	30 (9.9)	8 (2.6)	3 (1.0)

### 3.3. Univariate Analysis of WPV of Emergency Nurses

The study findings demonstrated that the scores pertaining to WPV were non‐normally distributed, necessitating the application of the rank‐sum test for subsequent statistical analysis. The results of the rank‐sum test revealed significant differences in WPV scores with respect to several factors: the number of years worked in the emergency department, professional title, work area, presence of family members accompanying patients, and the adequacy of nurse staffing levels (all *p* < 0.05). The results revealed that nurses with longer working experience in emergency, nursing technicians, and those with always enough nurse resources had the lowest WPV scores. Conversely, nurses working in the pre‐emergency area and those with sometimes enough (or seldom enough) nurse resources had the highest scores. Detailed information can be found in Table 4 (only variables with statistically significant differences are listed).

**TABLE 4:** Univariate analysis of WPV of emergency nurses.

Feature		Number	WPV (*M* (Q25, Q75))	Statistics	*p*
Working experience in emergency	1–2 years	61	2 (0, 8)	12.85	0.012^∗∗^
	3–5 years	36	3.5 (0, 7)		
	6–10 years	48	2 (0, 5)		
	10–15 years	108	1 (0, 3)		
	Over 15 years	51	1 (0, 3)		

Professional role	Nurse	33	5 (1, 9)	16.61	< 0.001^∗∗∗^
	Nursing technician	151	1 (0, 3)		
	Senior nursing technician	112	2 (0, 5)		
	Deputy chef nursing technician	8	1.5 (0, 2)		

Working area	Green area	91	1 (0, 3)	18.12	0.001^∗∗^
	Yellow area	38	2 (0, 3)		
	Red area	89	2 (0, 5)		
	EICU	80	0.5 (0, 4)		
	Pre‐emergency	6	7 (4.5, 8.75)		

Presence or absence of family accompaniment	Yes	243	2 (0, 4)	5932.50	0.013^∗^
	No	61	0 (0, 2)		

Nurse resource	Always enough	151	1 (0, 3)	19.47	< 0.001^∗∗∗^
	Sometimes enough	135	2 (0, 5)		
	Seldom enough	15	2 (0, 4.5)		
	Never enough	3	0 (0, 3)		

### 3.4. Correlation Between WPV, Resilience, and Moral Courage, and Their Dimensions Among Emergency Nurses

The results of the Spearman correlation analysis in this study showed that WPV was negatively correlated with moral courage, resilience, and their dimensions (*p* < 0.01). Additionally, moral courage and its dimensions were positively correlated with resilience and its dimensions (*p* < 0.01). For details, see Table 5.

**TABLE 5:** The correlation between WPV, resilience, and moral courage, and their dimensions among emergency nurses.

	Workplace violence	Moral courage	Morality	Empathy	Moral responsibility	Proposal of good care	Psychological resilience	Self‐improvement	Optimism	Strength
Workplace violence	1									
Moral courage	−0.296^∗∗^	1								
Morality	−0.263^∗∗^	0.909^∗∗^	1							
Empathy	−0.295^∗∗^	0.926^∗∗^	0.779^∗∗^	1						
Moral responsibility	−0.290^∗∗^	0.912^∗∗^	0.727^∗∗^	0.882^∗∗^	1					
Proposal of good care	−0.293^∗∗^	0.915^∗∗^	0.798^∗∗^	0.772^∗∗^	0.838^∗∗^	1				
Psychological resilience	−0.297^∗∗^	0.654^∗∗^	0.529^∗∗^	0.637^∗∗^	0.659^∗∗^	0.609^∗∗^	1			
Self‐improvement	−0.311^∗∗^	0.611^∗∗^	0.484^∗∗^	0.627^∗∗^	0.623^∗∗^	0.534^∗∗^	0.910^∗∗^	1		
Optimism	−0.251^∗∗^	0.552^∗∗^	0.441^∗∗^	0.537^∗∗^	0.559^∗∗^	0.525^∗∗^	0.868^∗∗^	0.723^∗∗^	1	
Strength	−0.272^∗∗^	0.643^∗∗^	0.524^∗∗^	0.611^∗∗^	0.645^∗∗^	0.617^∗∗^	0.967^∗∗^	0.810^∗∗^	0.809^∗∗^	1

^∗∗^ *p* < 0.01.

### 3.5. Multivariate Analysis of WPV of Emergency Nurses

The collinearity diagnostic outcomes demonstrated that all variance inflation factors (VIFs) were below 5, indicating the lack of multicollinearity among the variables and thereby permitting the execution of multiple linear regression analysis. In this analysis, the WPV score among emergency department nurses was set as the dependent variable, while statistically significant variables derived from univariate analysis (years of service in the emergency department, professional title, workplace, presence or absence of family member accompaniment during work, and adequacy of nurse staffing) and statistically significant variables from correlation analysis (specifically, moral courage and resilience scores) were designated as independent variables, and multiple linear regression analysis was carried out. The coding or assignment details of the independent variables are outlined in Table 6. The findings from the multiple linear regression analysis indicated that years of service in the emergency department, professional title, work area, adequacy of nurse staffing, moral courage, and psychological resilience were incorporated into the regression equation, acting as independent predictors of WPV among emergency department nurses (*p* < 0.05). The model is statistically significant (*F* = 28.205, *p* < 0.001), jointly explaining 35.0% of the variance in WPV among emergency department nurses. Refer to Table 7 for further details.

**TABLE 6:** The reference table of the baseline characteristics, moral courage, and psychological resilience.

Variables	Reference
Working experience in emergency	1–2 years of work as a reference (0, 0, 0, 0, 0); *X*1 = 3–5 (0, 1, 0, 0, 0); *X*2 = 6–10 (0, 0, 1, 0, 0); *X*3 = 10‐15 (0, 0, 0, 1, 0); *X*4 ≥ 15 (0, 0, 0, 0, 1)
Professional title	Nurse was settled as reference (0, 0, 0, 0); *X*1 = Nursing technician (0, 1, 0, 0); *X*2 = Senior nursing technician (0, 0, 1, 0); *X*3 = Deputy chef nursing technician (0, 0, 0, 1)
Working area	Green area was settled as reference (0, 0, 0, 0); *X*1 = Yellow area (0, 1, 0, 0, 0); *X*2 = Red area (0, 0, 1, 0, 0); *X*3 = EICU (0, 0, 0, 1, 0); *X*4 = Pre‐emergency (0, 0, 0, 0, 1)
Presence or absence of family	Yes = 1; no = 0
Nurse resource	Always enough = 1; sometimes enough = 2; seldom enough = 3; never enough = 4
Psychological resilience	Without transformation
Moral courage	Without transformation

**TABLE 7:** Multivariate analysis of WPV of emergency nurses.

	*B*	SE	Beta	*t* statistics	*p* value	VIF
Ref	10.246	0.889		11.531	< 0.001	
Working experience in emergency	−0.766	0.108	−0.331	−7.061	< 0.001	1.026
Professional title	−0.949	0.304	−0.148	−3.119	0.002	1.053
Pre‐emergency and green area	3.389	1.091	0.147	3.108	0.002	1.047
Nurse resource	0.558	0.24	0.111	2.33	0.02	1.051
Moral courage	−0.042	0.012	−0.225	−3.587	< 0.001	1.829
Psychological resilience	−0.034	0.01	−0.218	−3.431	< 0.001	1.882

*Note:* The statistical analysis yielded the following results: *F* = 28.205, *p* < 0.001, *R* ^2^ = 0.363, and adjusted *R* ^2^ = 0.350.

### 3.6. Construction and Evaluation of Structural Equation Model (SEM)

**FIGURE 1:**
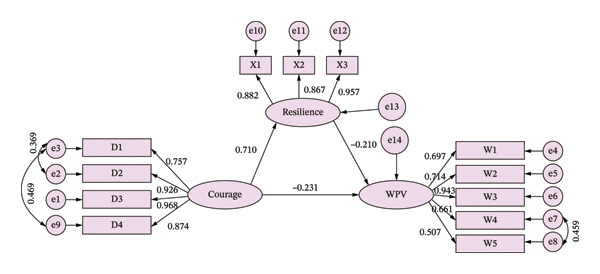
Structural equation model (standardized) of the mediating effect of psychological resilience between moral courage and workplace violence. *Note:* D1: morality, D2: empathy, D3: moral responsibility, D4: proposal of good care. X1: self‐improvement, X2: optimism, X3: strength, W1: physical assault, W2: verbal abuse, W3: intimidation and threats, W4: verbal harassment, W5: physical harassment.

This study revealed a significant correlation between WPV experienced by emergency department nurses and their levels of moral courage, psychological resilience, and the respective dimensions of these constructs. To delve deeper into the pathways and relative magnitudes of the factors influencing WPV in this context, we constructed a measurement model utilizing Amos 27.0. Within this model, moral courage, psychological resilience, and WPV were conceptualized as latent variables, whereas the dimensions of each scale functioned as observed variables. The model’s framework was established based on a comprehensive review of existing literature, theoretical underpinnings, and results from correlation analyses. The model’s goodness‐of‐fit was assessed using the maximum likelihood estimation technique. The initial iteration of the model produced a CMIN/DF ratio of 5.28 and an RMSEA value of 0.119. Following this, the model underwent refinement guided by modification indices (MIs). After incorporating three additional paths, the absolute fit index RMSEA improved to 0.078 (which is below the threshold of 0.08, indicating an acceptable fit), while the parsimony fit indices demonstrated a CMIN/DF ratio of 2.837, and the GFI, NFI, RFI, IFI, TLI, and CFI values all surpassed 0.9. All these indices conformed to the established fit criteria, as elaborated in Table 8. The standardized path coefficients of the refined model are visually represented in Figure 1.

**TABLE 8:** The revised fit indices for the mediation model of psychological resilience between moral courage and workplace violence.

Item	Measured value	Reference standards	Fitness evaluation
*X* ^2^/d*f*	2.837	1–3 is good, > 5 needs to be corrected	Meet demand

RMSEA	0.078	< 0.05 (good fit), < 0.08 (reasonable fit)	Meet demand

GFI	0.952	> 0.9	Meet demand
NFI	0.955		Meet demand
RFI	0.939		Meet demand
IFI	0.971		Meet demand
TLI	0.959		Meet demand
CFI	0.971		Meet demand

### 3.7. Path Analysis of the Model

The results of the path analysis on the influencing factors of WPV among emergency department nurses showed that moral courage and psychological resilience had direct and significant impacts on WPV. Moral courage had a positive effect on psychological resilience, with a path coefficient *β* = 0.710 (*t* = 15.347, *p* < 0.001). Psychological resilience negatively predicted WPV, with a path coefficient *β* = −0.210 (*t* = −2.466, *p* = 0.014). Additionally, moral courage negatively predicted WPV, with a path coefficient *β* = −0.231 (*t* = −2.709, *p* = 0.007). Detailed results are presented in Table 9.

**TABLE 9:** Pathway association hypothesis results (*n* = 304).

Path relationship	Standardized path coefficient	S.E.	C.R.	*p*
Psychological resilience ← moral courage	0.710	0.146	15.347	< 0.001
Workplace violence ← psychological resilience	−0.210	0.005	−2.446	0.014
Workplace violence ← moral courage	−0.231	0.014	−2.709	0.007

Abbreviations: C.R., *t* value; S.E., standard error.

### 3.8. The Mediating Role of Psychological Resilience on Moral Courage and WPV

The bootstrap method was employed in Amos 27.0 to test the mediating effect, with 5000 iterations conducted. Based on the test results, it can be observed that moral courage has a significant impact on WPV (*β* = −0.0755, *p* < 0.01), indicating that the total effect is established. In the bootstrap analysis, the indirect effect value is −0.0271, with a 95% confidence interval (−0.0507, −0.006). Since this confidence interval does not include 0, it suggests that the indirect effect is significant, and thus, the mediating effect is significant. The direct effect value is −0.0483, with a 95% confidence interval (−0.0739, −0.0228). As this confidence interval also does not include 0, it indicates partial mediation, with the mediating effect accounting for 35.89%. Detailed results are shown in Table 10.

**TABLE 10:** Results of the mediation effect test.

Path relationship	Effect relationship	Effect value	LLCI	ULCI	Ratio of effect	*p*
Moral courage ⟶ psychological resilience ⟶ workplace violence	Total effect	−0.0755	−0.0948	−0.0561		< 0.001
	Direct effect	−0.0483	−0.0739	−0.0228	64.11%	< 0.001
	Indirect effect	−0.0271	−0.0507	−0.006	35.89%	—

## 4. Discussion

This study represents the pioneering effort to demonstrate that the level of moral courage among emergency department nurses serves as a negative predictor of WPV. Furthermore, it has substantiated the mediating function of psychological resilience in the relationship between moral courage and WPV, with the mediating effect contributing to 35.89% of the total effect. This demonstrates that the moral courage of emergency department nurses not only directly influences the occurrence of WPV but also indirectly affects it through psychological resilience. Specifically, when nurses exhibit higher levels of moral courage, they can reduce the incidence of violent incidents by enhancing their psychological resilience.

Emergency nurses’ level of moral courage is negatively correlated with WPV and serves as an influencing factor. Nurses with higher moral courage scores tend to have lower WPV scores, indicating fewer incidents of WPV. Nurses with high moral courage possess a stronger sense of moral responsibility. When WPV occurs, they are more likely to adhere to ethical principles. This sense of principle and responsibility drives them to speak up for victims (e.g., stopping inappropriate behavior, reporting to superiors) even when facing professional risks such as retaliation, and to ensure the rationality of their professional conduct through continuous self‐reflection [37]. Moreover, nurses with higher moral courage exhibit greater compassion [38]. By utilizing effective communication and emotional resonance when addressing patients’ needs, they can mitigate conflicts stemming from misunderstandings or a lack of trust. Prior to the occurrence of a violent event, their acute insight allows them to promptly recognize and evaluate the severity of impending violence, intervene decisively at critical junctures, and resolutely halt aggressive behavior, thus effectively curtailing further conflict escalation [39]. These findings suggest that nursing managers should recognize the relationship between individual characteristics and WPV and organize regular training programs. For example, “Speak‐up” training [40]—which includes multisession lectures, hands‐on practice, and high‐fidelity simulation—can enhance nurses’ ability to actively advocate for colleagues and patients when facing violence. Such training not only improves nurses’ confidence and communication skills but also promotes their mental health. Additionally, video‐based scenario simulation [41] can recreate typical patient aggression scenarios, training nurses to recognize early warning signs of violence and follow standardized response procedures, thereby increasing their courage when confronting violent events. This type of intervention significantly improves nurses’ accuracy in assessing aggressive behavior and their willingness to report incidents, ultimately contributing to a safer and more harmonious working environment for healthcare workers.

Psychological resilience is negatively correlated with WPV and serves as a negative predictor of WPV. This finding is consistent with previous research results [24, 42]. Psychological resilience, serving as both a personality trait and an integral part of psychological capital, endows nurses with the capacity to navigate stressful scenarios, facilitating their effective transformation and adaptation to a diverse array of stress‐related challenges [42]. Nurses with higher levels of psychological resilience often possess stronger risk anticipation abilities [43]. This enables them to proactively apply their professional knowledge and experience to accurately identify potential risk factors for WPV and adopt preventive strategies to effectively avert the occurrence of violent incidents. This resilience is synonymous with adaptability and flexibility. Nurses with high levels of psychological resilience maintain an optimistic attitude and adopt proactive coping strategies when facing violent incidents. Following such experiences, they demonstrate stronger self‐regulation and recovery abilities [42], thereby alleviating the psychological distress and negative emotions induced by violence [44].

In addition, moral courage assumes a pivotal role in bolstering nurses’ psychological resilience [23]. Moral courage not only directly influences WPV but also indirectly affects it by enhancing psychological resilience. Specifically, nurses with higher levels of moral courage exhibit greater psychological resilience and experience fewer instances of WPV. Nurses demonstrating high moral courage display exceptional bravery and tenacity during violent incidents; they do not retreat in the face of threats but instead courageously step forward to protect their own rights, those of their colleagues, and the dignity of the nursing profession through concrete actions. This courage further strengthens nurses’ psychological adaptability, thereby elevating their psychological resilience. Conversely, robust psychological resilience enables nurses to remain calm and take appropriate actions—precisely reflecting the essence of moral courage [16]. Nurses with higher moral courage and psychological resilience are capable of effectively identifying violent incidents and utilizing their experience and professional skills to communicate and explain the situation to patients, thereby preventing the occurrence of violent incidents and protecting themselves from harm. Therefore, while addressing WPV, managers should also focus on cultivating nurses’ psychological resilience. For example, the “CARE” resilience promotion program [45] reduces anxiety levels caused by violence and alleviates tension by enhancing psychological resilience; the “Promoting Resilience in Nurses” program [46] delivers evidence‐based psychological resilience interventions to strengthen nurses’ ability to manage stress and improve mental health; and an online training program [47] provides web‐based courses and guided support to assess nurses’ acceptability of violence and changes in their psychological resilience before and after violent incidents, thereby enhancing their confidence and courage to cope with violence.

This study also conducted an in‐depth analysis of the current prevalence of WPV among emergency department nurses. The research data clearly indicated that 60.5% of emergency department nurses had encountered WPV within the past year, a result that aligns well with previous global reports on the incidence of WPV among nurses in this specific setting. Among the various types of violence, verbal abuse was the most common, accounting for 58.9% of cases. This form of violence included behaviors such as shouting, insults, and humiliation perpetrated by patients or their family members. Intimidation and threats followed, with an incidence rate of 36.5%, and physical assaults were reported at a rate of 26.6%. These findings closely resemble the results obtained in the study by Song et al. [48]. Patients admitted to the emergency department typically present with acute‐onset, life‐threatening conditions and a wide range of etiologies. Meanwhile, their family members often harbor a mindset characterized by an intense eagerness for medical treatment and excessively high expectations. When faced with prolonged waiting times or unsatisfactory treatment outcomes, their anxiety can quickly escalate into accusations or even physical attacks on nurses. This dynamic significantly heightens the risk of conflicts between family members and emergency healthcare providers, ultimately contributing to the high frequency of violent incidents.

Moreover, this study identified several demographic factors that are significantly associated with the occurrence of WPV among emergency department nurses. These factors encompass the number of years worked in the emergency department, professional title, work area, and the adequacy of nurse staffing (*p* < 0.05). For example, nurses with greater experience and higher professional titles tend to score lower on the WPV scale. This may be attributed to their extensive clinical expertise, deeper understanding of the emergency department’s unique environment and patient needs, and enhanced ability to anticipate emotional fluctuations and potential conflicts among patients and their families [49]. Additionally, their higher professional competence and richer clinical experience strengthen their resilience against WPV [50]. Conversely, inadequate nurse staffing and working at the triage desk can subject nurses to increased work pressure and burdens, thereby further elevating the risk of WPV.

Our research underscores the influence of nurses’ personal traits, specifically moral courage and psychological resilience, on WPV. Although the data were derived from emergency departments in China, moral courage, as an intrinsic quality grounded in personal moral principles, possesses cross‐cultural universality. Accordingly, the “moral courage ⟶ psychological resilience ⟶ WPV” pathway model proposed in this study is expected to serve as a reference for nursing management practices worldwide, particularly in emergency care settings that face similarly high risks of violence exposure. Nursing managers across different countries and regions can utilize this model to design targeted intervention programs tailored to their local legal environments, organizational cultures, and violence management policies. For instance, moral courage can be strengthened through ethical decision‐making simulation training, while psychological resilience can be enhanced via group‐based resilience training programs. Such interventions may effectively reduce the incidence of WPV, safeguard nurses’ legitimate rights and interests, and promote the stable and sustainable development of the nursing workforce.

## 5. Limitation

First, this study employed a cross‐sectional design, which cannot establish causality. Future research could adopt longitudinal or experimental designs to validate our conclusions. Second, this survey employs self‐report measures, and the violence scale may carry the risk of inducing recall/report bias. The Nursing Moral Courage Scale (NMCS), whose items address sensitivity to moral principles, may exhibit social expectation bias. Respondents may tend to report behaviors or attitudes that better align with professional ethics standards, thereby overestimating their own level of moral courage. Although we implemented measures like anonymous data collection and independent responses during data gathering to minimize this bias, residual social expectation effects cannot be entirely ruled out. Further research should incorporate multiple information sources and/or various data collection methods to overcome the subjective biases associated with self‐reporting. Third, although this study was a multicenter investigation (covering five tertiary Grade A hospitals in Liaoning Province), the sample was still limited to emergency department nurses from a single province. Whether the findings represent the national or regional context requires further validation; future studies could expand the sample size to confirm these results. Finally, this study did not measure organizational culture across hospitals (e.g., leadership support, ethical climate), factors that may interact with nurses’ levels of moral courage and psychological resilience, thereby confounding WPV outcomes. Future research should incorporate additional factors for comprehensive analysis.

## 6. Conclusion

This study developed an SEM to investigate WPV among emergency department nurses. Specifically, it examined the action pathways and underlying influence mechanisms through which moral courage and psychological resilience of emergency department nurses affect WPV. Additionally, the study explored the mediating role played by psychological resilience in these relationships.

## 7. Implications for Nursing Management

WPV stands as one of the foremost occupational hazards within healthcare institutions across the globe, exerting a profound influence on nurses’ career progression. Through our research, we have elucidated the pivotal mediating role that psychological resilience plays in this scenario, thereby presenting a fresh vantage point for the development and refinement of intervention strategies aimed at mitigating WPV. It is imperative for nursing managers to take full account of and actively foster the cultivation of nurses’ individual moral courage and psychological resilience. By doing so, nurses will be better equipped to effectively manage and respond to instances of WPV, all the while preserving a sound mental state. Ultimately, this approach will facilitate the concurrent improvement of work efficiency and job satisfaction among nursing staff.

## Author Contributions

Kai Liu and XinYue Zhang contributed equally to this work and share the first authorship. Kai Liu and XinYue Zhang made substantial contributions to conception and design, or acquisition of data, or analysis and interpretation of data. Kai Liu and XinYue Zhang were involved in drafting the manuscript or revising it critically for important intellectual content. XiangHong Sun and Ling Fan have given final approval of the version to be published. Each author should have participated sufficiently in the work to take public responsibility for appropriate portions of the content. XiangHong Sun and Ling Fan agreed to be accountable for all aspects of the work in ensuring that questions related to the accuracy or integrity of any part of the work are appropriately investigated and resolved.

## Funding

The present study received no external funding.

## Conflicts of Interest

The authors declare no conflicts of interest.

## Data Availability

The data that support the findings of this study are available from the corresponding author upon reasonable request.
